# Control of ROS production and T-cell turnover by HTLV-p13

**DOI:** 10.1186/1742-4690-8-S1-A154

**Published:** 2011-06-06

**Authors:** Micol Silic-Benussi, Ilaria Cavallari, Luigi Chieco-Bianchi, Fabio di Lisa, Donna M D’Agostino, Paolo Bernardi, Vincenzo Ciminale

**Affiliations:** 1 Department of Oncology and Surgical Sciences, University of Padova, I-35128 Padova, Italy; 2 Consiglio Nazionale delle Ricerche Institute of Neuroscience at the Department of Biomedical Sciences, University of Padova, I-35121 Padova, Italy

## 

The present study was aimed at gaining insight into the function of p13, an 87-amino acid mitochondrial protein expressed by HTLV-1. Although necessary for viral propagation in vivo, the mechanism of p13 function is incompletely understood. In previous studies we showed that p13 exerts antitumor effects in experimental transformation models. More recently, using synthetic p13 and isolated mitochondria, we showed that the protein triggers an inward K+ current that leads to mitochondrial depolarization, increased activity of the respiratory chain, and reactive oxygen species (ROS) production. These findings prompted us to test the effects of p13 on ROS in living cells, including T-cells, the main targets of HTLV-1 infection in vivo.

Expression of p13 in primary T-cells resulted in cell activation, measured using the CD38 surface marker. p13-induced activation was blocked in the presence of ROS scavengers and was not observed using a p13 mutant that was inactive in the in vitro assays, indicating a connection between the effects on ROS those on mitochondrial K+ influx. In the context of the transformed cell line Jurkat, p13 did not affect ROS levels unless the cells were subjected to glucose deprivation, which led to a p13-dependent increase in ROS and cell death. Using RNA interference we confirmed that expression of p13 also influences glucose starvation-induced cell death in HTLV-1-infected cells. Taken together, our findings indicate that in the context of the HTLV-1 propagation strategy, p13 could increase the pool of "normal" infected cells while culling cells acquiring a transformed phenotype, thus favoring life-long persistence of the virus in the host.

